# An evaluation of the isoniazid preventive therapy program performance for under-fives in Kwekwe City, January 2019 - December 2020: a descriptive cross-sectional study

**DOI:** 10.11604/pamj.2022.42.104.33276

**Published:** 2022-06-08

**Authors:** Nyashadzashe Cosmas Makova, Mary Muchekeza, Joconiah Chirenda, Addmore Chadambuka, Emmanuel Govha, Tsitsi Patience Juru, Notion Tafara Gombe, Mufuta Tshimanga

**Affiliations:** 1 Department of Primary Health Care Sciences, University of Zimbabwe, Harare, Zimbabwe,; 2 Kwekwe City Council Department of Health, Kwekwe, Zimbabwe,; 3 Department of Surgical Care Sciences, University of Zimbabwe, Harare, Zimbabwe,; 4 African Field Epidemiology Network, Harare, Zimbabwe

**Keywords:** Isoniazid preventive therapy, program evaluation, Kwekwe, Midlands Province, tuberculosis

## Abstract

Childhood tuberculosis (TB) is underserved in resource-constrained endemic areas. Zimbabwe National Tuberculosis Program recommends tuberculosis prevention treatment for children aged <5 years who are close contacts of smear-positive TB cases. The Isoniazid Preventive Therapy (IPT) program performance had never been evaluated since its inception in 2010. We therefore, assessed the IPT program's inputs, processes, outputs, and outcomes. We conducted a process evaluation using the logic model in Kwekwe City. We recruited twenty-seven health care workers from all the five municipal health facilities. Smear-positive guardians of under 5 children, health care workers, and registers were the study population. Data were collected using a questionnaire and checklists and presented as frequencies and proportions. The IPT program met requirements in provision of guidelines (10/10), screening tools (15/15) and on-the-job trainings done in all five health facilities. Isoniazid tablets supply and quarterly budgeting did not meet meeting program requirements. Fifty-nine out of 231 (25.5%) children contacts of sputum-positive TB patients were screened. Fifty-one of the 59 (86.4%) children were initiated on IPT, 42/51 (82.4%) completed the course, one developed TB, 3/51 were still on treatment and 5/51 were lost to follow up. No dropouts and deaths were recorded. Unavailability of drugs was a barrier to the IPT and negatively impacts the TB elimination program. Contact screening was the bottleneck in the successful implementation of the program. Adequate staff and provision of drugs might improve the program. We recommended the recruitment of more healthcare workers and the budget for the program.

## Introduction

Tuberculosis (TB) is a communicable disease that can be treated and prevented. It is a major cause of illness, one of the top ten causes of death globally, and the leading cause of death caused by a single infectious agent [1]. Between 2015 and 2030, the End TB Strategy set targets of a 90% reduction in TB deaths and an 80% reduction in the TB incidence rate (new and relapse cases per 100 000 population per year). The 2020 milestones were reductions of 35% and 20%, respectively [1]. Tuberculosis control remains a global challenge, especially in resource-limited countries such as Zimbabwe, where the estimated TB incidence was 221/100,000 in 2017 [2,3]. Every year, tens of millions of children are exposed to Mycobacterium tuberculosis (MTB), and TB remains a leading infectious cause of childhood morbidity and mortality worldwide [4]. Exposure to the MTB bacteria leads to TB infection, which can remain dormant for years, but it can also progress to active TB [5]. Childhood tuberculosis is underserved in resource-constrained endemic areas because children are thought to develop mild forms of disease and contribute little to the disease transmission [6]. However, children contribute a significant proportion of the disease burden and suffer severe tuberculosis-related morbidity and mortality, particularly in endemic areas [7-10].

Young children are usually infected with MTB by an infectious adult TB case [11,12]. In the absence of appropriate preventive therapy, young children are at high risk of primary disease progression and disseminated disease after MTB infection [11-14]. Six months of Isoniazid Preventive Therapy (IPT) in MTB-infected individuals reduces the risk of developing TB by 60-65% over 5 years follow-up in all age groups. The World Health Organization (WHO) and Zimbabwe´s National TB Programme recommend IPT for all children aged <5 years who are in close contact with an infectious (smear- and/or culture-positive) TB case [15,16]. Without any intervention, 5-10% of infected children will develop active TB within one year, and the risk is the highest among the youngest (<2 years old) or HIV-infected children [17].

Isoniazid preventive therapy is safe and well tolerated by children; major serious adverse events, such as hepatotoxicity and pyridoxine deficiency, are uncommon in children [18,19]. Its efficacy in preventing disease is as high as 90% when taken correctly [12]. With good adherence, isoniazid monotherapy is effective in preventing incident TB in children infected with susceptible strains of MTB [16]. According to modelling studies, preventive measures must be included in TB programs in order to achieve TB eradication by 2050 [14]. It is estimated that up to 43% of infected children aged 12 months and 24% of those aged 1-5 years develop TB disease [12]. Despite recommendations to provide isoniazid preventive therapy (IPT) to eligible children aged <5 years who are in close contact with an infectious tuberculosis (TB) case, IPT delivery in high-burden tuberculosis settings remains poor [2]. Kwekwe City started implementing the IPT program in 2010 in all of the City Council´s health facilities. The IPT program data indicated that not all clients eligible for IPT were initiated on the IPT (23 - 35% for period 2016 to 2018) against a target of 100% which is recommended by the Zimbabwe national TB program. Having noted the challenges which were being experienced in the IPT program, we evaluated the inputs, processes, outputs, and outcomes for the IPT program for children under 5 years in Kwekwe City and reasons for failure.

## Methods

**Study design:** we conducted a process evaluation using the logic model in Kwekwe City.

**Study setting:** the study was conducted in all five health facilities in Kwekwe City. These are Amaveni Clinic, Al Davies Clinic, Mbizo 1 Clinic, Mbizo 11 Clinic and Mbizo 16 Clinic. According to the Zimbabwe National Statistics Agency (ZIMSTATS) Midlands Province District Population Projections report of 2020, the current population of Kwekwe City is approximately 117,116. The majority of Kwekwe City's residents are artisanal miners. Areas with artisanal mining activities and poverty have been identified as TB hotspots.

**Study population:** the healthcare workers who include nurses and doctor at all the five health facilities in Kwekwe City were recruited into the study. Tuberculosis focal person, Assistant Director Health (Nursing) and Assistant Director Health (Environment), Microscopist, and the Kwekwe district TB focal person were recruited as key informants. We also recruited child-guardian pairs when they attended the TB clinic. Tuberculosis treatment and IPT registers were reviewed for data on patients with child contacts, the number of the child contacts under 5 that were listed, the number of children that were screened, and the number of children under 5 who were put on IPT, the number of children who developed tuberculosis whilst on IPT and number of children who completed treatment.

**Sampling:** all the health facilities offering IPT in Kwekwe City were included in the study. The nurses at OI/ART and IMNCI departments were randomly sampled. The TB focal person, Assistant Director Health (Nursing) and Assistant Director Health (Environment), Microscopist, the Kwekwe district TB focal person were purposively sampled into the study as key informants. We conveniently sampled selected clients at the TB clinic who had contacts less than the age of 5 years to be in the focus group discussion.

**Sample size calculation:** using Dobson formula:


$$n=\frac{{\left(Z_{\alpha /2}\right)}^{2}pq}{\delta^{2}}$$

Where Zα/2=1.96, p=0.023, 6 out of 368 (1.6%) children developed evidence of new TB disease and required definitive anti-tuberculosis therapy whilst on IPT, (Okwara *et al*. 2017, Kenya) [20], delta is 0.05, confidence interval is 95%, non-response rate is 10%, a minimum sample size of 27 health workers was calculated. We recruited eight caregivers into the focus group discussion.

**Data collection:** an interviewer-administered questionnaire was used to elicit information on operations of the program, reasons for low coverage of IPT initiation from study participants. Key informant interviews were done to gather information about the number of health workers trained in IPT and the reasons for the failure of the TB preventative treatment program in Kwekwe City. A focus group discussion was conducted to gather information on the reasons for the failure of the program from the clients. Participant bias occurs when participants respond to questions based on what they believe is the correct answer or what is socially acceptable rather than what they truly feel or do. To minimize participant bias during focus groups, we framed open-ended questions to prevent participants from simply agreeing or disagreeing, and we guided them to provide truthful and honest answers. If the answers did not seem correct, we asked the question again in a different way. Inputs are the resources and materials invested in the program. Some of the inputs that were assessed included staff, money, vehicles, drugs, treatment and policy guidelines, TB manuals, and TB health promotion materials. Processes are the activities conducted utilizing the inputs provided for the program. Some of the processes that were assessed included planning and budgeting, review meetings, support and supervision, advocacy and community sensitization, and staff training. Outputs are short-term logical results of implementing program activities. Output indicators included the number of children contacts under the age of 5 years eligible for screening, number of children screened, number eligible for IPT, number of Information Education Communication (IEC) materials on IPT distributed, and number of IPT advocacy and sensitization meetings conducted. Program outcomes are medium-term logical consequences/results of achieving several outputs. The TB-IPT program outcome indicators included the number of children eligible for IPT initiated on IPT, number of children who completed IPT, number of dropouts due to toxicity, and number of children who developed TB during IPT. These were assessed through a structured interviewer-administered questionnaire, review of records, and checklists.

**Data analysis:** we used Epi Info 7.2.4.0™ (CDC, 2020) statistical package to capture and analyse data from questionnaires. Frequencies, proportions, and medians were calculated using the same software. We analysed qualitative data from checklists and key informants manually. Key themes and quotes on the reasons for the underperformance of the IPT program were identified and reported.

**Ethical considerations:** we obtained permission to conduct the study from the director of health services of Kwekwe City and the Health Studies Office (HSO). We obtained informed written consent from all the interviewees and we assured them of confidentiality. To ensure confidentiality, we stored the completed forms in a locked cabinet, and electronic records were stored in a password-protected computer. All information that would identify a participant was not included. Since data collection took place during the COVID-19 pandemic, the essential precautions were strictly adhered to by ensuring social distancing between the interviewer and study participants, hand hygiene, and wearing masks that adequately covered the nose and mouth.

## Results

We visited all five health facilities. We recruited 27 participants (21 nurses, and one of the following, environmental health officer, TB coordinator, doctor, Microscopist, assistant director of health (nursing) and assistant director of health (environment)), of these, 20 were females. The median age was 48 (Q_1_= 36; Q_3_=53) years and the median years in service was 20 (Q_1_= 10; Q_3_=30). Three of the eight participants for the FGD were males and the median age was 34 (Q_1_= 29.5; Q_3_=39).

**Figure 1:**
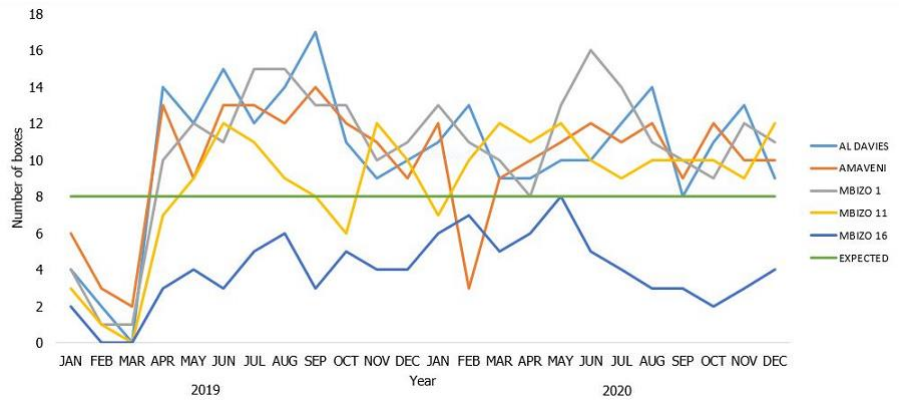
distribution of isoniazid by facility by month in Kwekwe City, 2019 - 2020

**Inputs used to run the program:** the city had adequate (10/10) healthcare worker desk guides for the management of tuberculosis and (15/15) TB screening tools that were extracted from the guidelines printed and displayed. However, Kwekwe City did not draft, print or distribute any IEC materials on the IPT program for children under 5 (Table 1). The city experienced a stock out isoniazid 100mg tablets in the first quarter of 2019 between January and March (Figure 1). Kwekwe City had 54 of the 65 required nurses and relied heavily on locum nurses. There were no employed community health workers or environmental health technicians during the period under review.

**Table 1:** inputs used to run the IPT program, Kwekwe City, 2019 - 2020

Item	Jan-Dec 2019	Target	Jan-Dec 2020	Target
IPT guidelines	10	10	10	10
TB screening tools	15	15	15	15
Vehicles	1	1	1	1
Motor cycles	0	1	0	1
IEC materials	0	1000	0	1000
Nurses	54	65	54	65

**Processes involved in running the IPT program in Kwekwe City:** no quarterly planning and budgeting were done by Kwekwe City for the TB program and no quarterly meetings were held during the period. There were on-the-job trainings done in all health facilities between 2019 and 2020, 54 (100%) of the nurses were trained in the first quarters. Each of the five health facilities in Kwekwe City had training sessions done during the first quarters of 2019 and 2020 and the nurses were the participants. Furthermore, no advocacy and community sensitization were done in 2019 and 2020. However, support and supervision were done monthly at the health facilities.

**Outputs of the IPT program in Kwekwe City:** the pulmonary tuberculosis (PTB) index case to child contact ratio was 1: 0.88. From January 2019 to December 2020, 231 children contacts were elicited and all were eligible for screening. Kwekwe City had screened 59/231 (25.5%) children under the age of 5 who were contacts of sputum positive TB patients, and all the 59 children were eligible for IPT. None of the health facilities did community sensitization on IPT (Table 2).

**Table 2:** outputs of the IPT program in Kwekwe City, 2019 - 2020

Indicator	Jan-Dec 2019	Target	Jan-Dec 2020	Target	Cumulative Jan 2019- Dec 2020
TB clinic burden	129	-	134	-	263
No. of under 5 children contacts eligible for screening	116	-	115	-	231
No. of children under 5 screened	33 (28.4%)	116 (100%)	26 (22.6%)	115 (100%)	59 (25.5%)
No. eligible for IPT	33	33	26	26	59
No. of IEC materials on IPT distributed	0	10 000	0	10 000	0
No. of IPT advocacy and sensitization meetings	0	12	0	12	0

**Figure 2:**
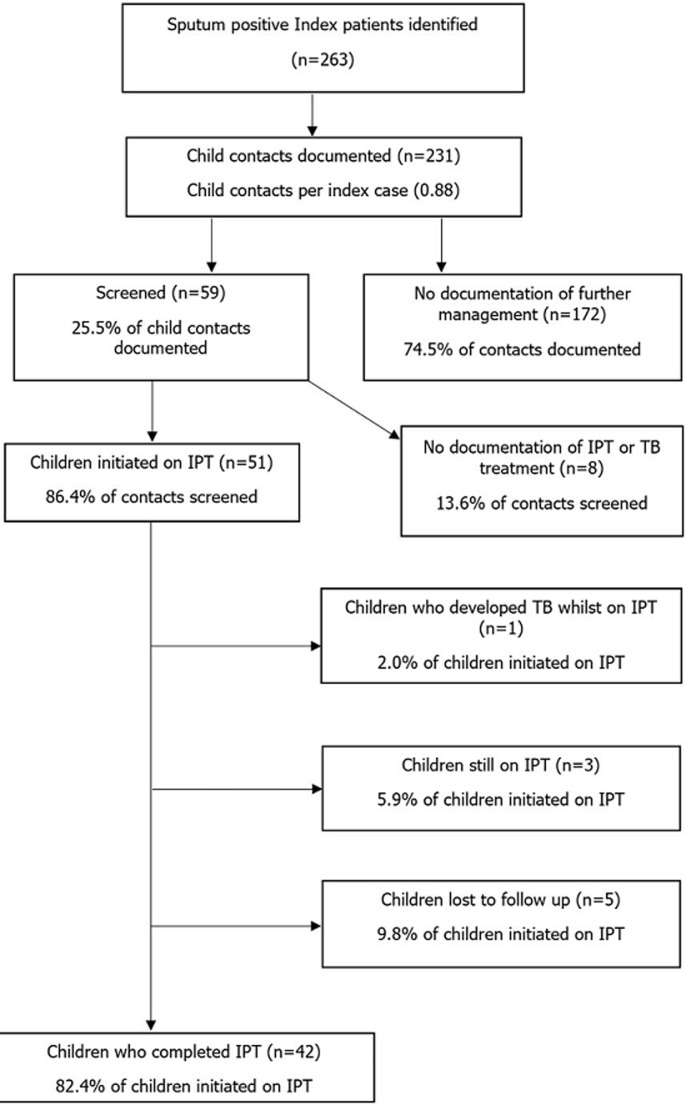
summary of the IPT program results in Kwekwe City, 2019 - 2020

**Outcomes of the IPT Program in Kwekwe City:** a total of 231 children under the age of five were contacts of sputum-positive cases. Of the 231, only 59 were screened. Of the 59 children who were screened and eligible for IPT, Kwekwe City started 51 (86.4%) of children on IPT. A total of 42/51 children completed the INH six-month course. Five, 5/51 children were lost to follow-up. There were no dropouts due to drug toxicity and no deaths were recorded (Table 3). Figure 2 summarizes the IPT program for children under the age of 5 years in Kwekwe City.

**Table 3:** outcomes of the IPT program in Kwekwe City, 2019 - 2020

Indicator	Jan-Dec 2019	Target	Jan-Dec 2020	Target	Cumulative Jan 2019- Dec 2020
No. of children under 5 initiated on INH	29 (87.9%)	33 (100%)	22 (84.6%)	26 (100%)	51 (86.4%)
No. of children who developed TB during IPT	1	0	0	0	1
No. of children who completed IPT	24(82.8%)	29	18(81.8%)	22	42(82.4%)
Number of children still on IPT	0	0	3	3	3
Number of children on IPT lost to follow up	4	0	1	0	5

**Reasons for the poor performance of the program:** the key informants mentioned lack of skill and confidence to perform gastric washings as one of the reasons why the gastric washings are not being done. One key informant said, “*Some of the healthcare workers always give an excuse when they are asked to perform gastric washings and this can be a lack of confidence on their part*.” Lack of resources was also indicated as a reason for limited screening as the council clinics did not have the reagents to perform a tuberculin skin test. “*The X-ray machine at Kwekwe General Hospital was sometimes not functional and most people who were referred to the private facilities did not go because of the prices*,” said another key informant. Lack of registers was mentioned as a reason for poor documentation as health facilities ended up improvising and this could also affect the follow-up processes. Furthermore, the employment of a lot of locum nurses in the city was reported to be contributing to poor documentation as they did not carry the responsibility to document.

The clients noted that they did not know about TB preventative therapy for children under the age of 5 years. Some said it was the first time they had heard about preventative therapy for children as one participant said, “*I have been taking my medication at this facility for two months now and I am only hearing about medicine to prevent TB in children now*.” Poor knowledge and fear of stigma were also observed as one client said, “*It is really difficult for me to bring my child here, I am also on HIV treatment and I know what we face in the community. I don´t want my child to go through the same. They don´t have HIV so they are fine*.”

## Discussion

We evaluated the inputs, processes, outputs, and outcomes of the IPT program for children under the age of five in Kwekwe City, as well as the reasons for failure. We found that the program lacked resources, which hampered the processes and ultimately reduced the outputs and outcomes. Lack of skill and confidence in performing gastric washings contributed to the program's failure to screen and test patients, resulting in the program's failure. The World Health Organisation recommends that certain criteria for staffing levels should be met before the program is ready to proceed with the addition of contact screening and management. The availability of adequate human capital is the backbone of any public health program because they are the executors of the activities required to run the program or project. We found that Kwekwe City faced human capital challenges between the period January 2019 - December 2020 as they did not meet the WHO recommendations required by the WHO of 4.45 health workers per 1000 population needed to achieve 80% coverage for selected SDG tracer indicators [21]. Having low numbers of health workers in the program increases the workload to the few available and will negatively affect the deliverables of the program. The study findings were in keeping with the findings in Shurugwi District, Zimbabwe, where the number of health workers who were involved in the IPT program was below the optimum required [22]. Similarly, in Mozambique, it was found that a lack of resources, including personnel, within the national TB control program was one of the potential factors contributing to the poor quality of the TB care cascade [23].

Kwekwe City faced pyridoxine and isoniazid stock-outs in 2020 leading to clients not being started on this preventive therapy. The unavailability of drugs is a barrier to tuberculosis preventative treatment, and it will negatively impact the TB elimination program. The INH stock-outs may also contribute to low confidence in the healthcare workers about the IPT program. The drug stock-outs could have been because of logistical challenges nationally. The findings were similar to those in Bhopal, India, where the key barriers associated with the implementation of IPT among the child contacts at the program level included the unavailability of drugs [24].

For the period under review, Kwekwe City never received IEC materials in terms of pamphlets or posters to educate health workers, clients, and the community at large. Information Education Communication materials provide information and education about IPT and should be placed in strategic locations where members of the community can access them, such as beer halls, shopping malls, churches, clinics, and schools. Improving community education through the distribution of IEC materials on the benefits of IPT may improve program uptake. According to the WHO Guidance for National Tuberculosis Programs on the Management of Tuberculosis in Children, to effectively implement IPT and monitor this activity, all health staff must be adequately trained and sensitized on the importance of contact screening, and they must be effective in communicating this information to the community [15].

Kwekwe City did not conduct advocacy or community sensitization for program success, resulting in low program uptake. Local advocacy efforts are aimed at increasing public support for the IPT program. Even when the IPT program began, the city did not conduct advocacy or community sensitization. We concluded that there was a need to advocate for the program by involving local community leaders and the community at large in program implementation to increase program uptake. Du Preez concluded that opportunities for initiating chemoprophylaxis in vulnerable children following TB exposure are frequently missed, and that awareness of the importance of chemoprophylaxis in young and HIV-infected children should be increased among healthcare workers and the general public [25]. Only a quarter of the elicited contacts were screened in Kwekwe City, which could be attributed to a lack of planning and budgeting, insufficient staffing levels, a lack of formal training among health care workers, a lack of transportation, and a lack of community sensitization. When screening is inadequate, a significant proportion of children under the age of five who require IPT do not receive it. Ending the TB epidemic requires not only curing the disease but also preventing it from starting in the first place. Of the 525 child contacts identified, in a study done in Cape Town, South Africa, less than half were screened showing that contact tracing and screening remain a problem in areas with high TB burden [16]. Similar findings were found in the study by Wyk [26]. Furthermore, high-burden countries have reported extremely poor compliance with screening and initiation of IPT.

We found a PTB index case to child contact ratio of 1: 0.88. This ratio was lower than that recommended by the WHO TB treatment guidelines which state that for every TB index case diagnosed, at least one child contact was aged less than 5 years (1: 1). The reason behind this could have been due to poor documentation of the registers which was noted during the review of records. Poor documentation affects decision making and interventions made from poor data might be inappropriate. The ratio was lower than that found in Ethiopia where the ratio was 1: 1.32 [27] but higher compared to that in Nelson Mandela Bay Health District, Eastern Cape Province, South Africa where 0.53 child contacts were identified per index patient [28]. During the period under study, a tenth of the children who were on IPT was lost to follow up and that´s a significant proportion. The children could have been lost to follow up due to drug shortages and also poor documentation of the registers. Losing clients will negatively impact the effectiveness of the program. Ending tuberculosis (TB) epidemic by 2030 requires identification and treatment of latent TB infection to prevent progression to active disease thus losing clients to follow-up will affect the treatment of latent disease. In our study, about four-fifths of children who were initiated on IPT completed the recommended IPT treatment. The IPT completion rate in this study was much higher than that reported in South Africa in Nelson Mandela Bay Health District, Eastern Cape Province, where only 10 (9.3%) children completed the 24-week IPT course [28]. Even though the treatment completion rate was high, it still needs a great effort to score the 100% completion rate.

**Limitations of the study:** our study had some limitations. Since we conducted one focus group discussion, results may not be generalizable. Participant bias occurs when participants respond to questions based on what they believe is the correct answer or what is socially acceptable rather than what they truly feel or do. To minimize participant bias, we framed open-ended questions to prevent participants from simply agreeing or disagreeing, and we guided them to provide truthful and honest answers. If the answers did not seem correct, we asked the question again in a different way. To minimise interviewer bias, we used a well-structured questionnaire for all participants in the comfort of their workplaces.

## Conclusion

In Kwekwe City, the implementation of the IPT program was hampered by a lack of resources, a lack of planning, and poor contact screening. The IPT program for children under the age of five in Kwekwe City is underperforming, with only one-quarter of the children contacts screened. Contact screening was the stumbling block to the program's successful implementation. There were challenges with disseminating information to community members, who are key stakeholders in the program's success. We recommended the production and distribution of IEC material on the IPT program and intensified advocacy, communication, community education, and social mobilization. We also recommended the recruitment of health care workers that is environmental health technicians and community health workers.

**Public health actions taken:** we gave health education to the study participants and emphasis was given on the need for screening clients as this was vital to the success of the program. We also emphasized the importance of documentation in registers.
